# IRE1-Mediated Unfolded Protein Response Promotes the Replication of Tick-Borne Flaviviruses in a Virus and Cell-Type Dependent Manner

**DOI:** 10.3390/v13112164

**Published:** 2021-10-27

**Authors:** Veronika J. M. Breitkopf, Gerhard Dobler, Peter Claus, Hassan Y. Naim, Imke Steffen

**Affiliations:** 1 Department of Biochemistry, University of Veterinary Medicine, 30559 Hannover, Germany; veronika.breitkopf@tiho-hannover.de (V.J.M.B.); hassan.naim@tiho-hannover.de (H.Y.N.); 2 Research Center for Emerging Infections and Zoonoses, University of Veterinary Medicine, 30559 Hannover, Germany; 3 German Center of Infection Research (DZIF), Bundeswehr Institute of Microbiology, 80937 Munich, Germany; gerharddobler@bundeswehr.org; 4 SMATHERIA gGmbH—Non-Profit Biomedical Research Institute and Center for Systems Neuroscience (ZSN), 30625 Hannover, Germany; peter.claus@smatheria.org

**Keywords:** flavivirus, tick-borne encephalitis virus, Langat virus, viral replication, ER stress, unfolded protein response, neuroinfection

## Abstract

Tick-borne flaviviruses (TBFV) can cause severe neurological complications in humans, but differences in tissue tropism and pathogenicity have been described for individual virus strains. Viral protein synthesis leads to the induction of the unfolded protein response (UPR) within infected cells. The IRE1 pathway has been hypothesized to support flavivirus replication by increasing protein and lipid biogenesis. Here, we investigated the role of the UPR in TBFV infection in human astrocytes, neuronal and intestinal cell lines that had been infected with tick-borne encephalitis virus (TBEV) strains Neudoerfl and MucAr-HB-171/11 as well as Langat virus (LGTV). Both TBEV strains replicated better than LGTV in central nervous system (CNS) cells. TBEV strain MucAr-HB-171/11, which is associated with gastrointestinal symptoms, replicated best in intestinal cells. All three viruses activated the inositol-requiring enzyme 1 (IRE1) pathway via the X-box binding protein 1 (XBP1). Interestingly, the neurotropic TBEV strain Neudoerfl induced a strong upregulation of XBP1 in all cell types, but with faster kinetics in CNS cells. In contrast, TBEV strain MucAr-HB-171/11 failed to activate the IRE1 pathway in astrocytes. The low pathogenic LGTV led to a mild induction of IRE1 signaling in astrocytes and intestinal cells. When cells were treated with IRE1 inhibitors prior to infection, TBFV replication in astrocytes was significantly reduced. This confirms a supporting role of the IRE1 pathway for TBFV infection in relevant viral target cells and suggests a correlation between viral tissue tropism and the cell-type dependent induction of the unfolded protein response.

## 1. Introduction

Tick-borne encephalitis virus (TBEV) belongs to the genus *Flavivirus* within the family of *Flaviviridae*. It is mainly transmitted by ticks, but also alimentary infection by the consumption of raw milk products from infected ruminants is well-known. Despite an available vaccine, increasing cases of TBEV infections were registered during the last years [1]. Upon crossing the blood-brain barrier, TBEV can cause severe neurological inflammation and in some cases even long-term neurological sequelae [2]. Individual TBEV strains have been described to show differences in pathogenicity [3,4,5]. TBEV comprises a single-stranded, positive-sense RNA genome of about 11 kb in length. The genome is translated into a single polyprotein that is co- and post-translationally cleaved by viral and cellular proteases into ten individual proteins, including three structural proteins (C, prM, E) and seven nonstructural proteins (NS1, NS2A, NS2B, NS3, NS4A, NS4B, NS5) [6]. Viral RNA replication, translation, and virus assembly is associated with endoplasmic reticulum (ER) membranes [7,8]. Flavivirus infection alters the structure of the ER, causing proliferation of the ER membranes that facilitate flavivirus protein synthesis [7,9]. The increased amount of viral proteins synthesized results in the accumulation of unfolded and misfolded proteins in the ER lumen [10]. The resulting imbalance between the protein-folding load and the capacity of the ER causes ER stress within the infected cells. To restore protein-folding homeostasis, the cells activate intracellular signaling pathways referred to as the unfolded protein response (UPR) [11].

In mammals, the UPR pathways are initiated by three stress sensors located in the ER membrane: inositol-requiring enzyme 1 (IRE1), protein kinase R-like ER kinase (PERK) and activating transcription factor 6 (ATF6) (reviewed in [12]). Under normal conditions, the sensors are maintained in an inactive state by association with the ER chaperone binding immunoglobulin protein (BiP). Upon ER stress, BiP dissociates from the stress sensors owing to the higher affinity for misfolded proteins [13]. Subsequently, the sensors are activated and downstream signaling is initiated. In ER stress conditions, the kinase domain of IRE1 autophosphorylates and the endoribonuclease (RNase) domain becomes activated [12,13]. Subsequently, the RNase domain splices the X-box-binding protein 1 (XBP1) mRNA, enabling the translation of the active transcription factor XBP1s [14,15]. XBP1s induces the transcription of target genes that are involved in the expression of chaperones and lipid synthesis [14,16,17] resulting in an increased folding capacity and an expansion of the ER membrane. Upon activation, PERK phosphorylates the α subunit of eukaryotic translation initiation factor 2 (eIF2α) resulting in p-eIF2α and leading to a transient attenuation of mRNA translation [16,17]. When ATF6 is released from BiP, it translocates to the Golgi apparatus, where it is cleaved proteolytically [18,19]. The resulting p50 fragment (ATF6f) migrates to the nucleus where it upregulates different target genes [20,21].

Several flaviviruses have been shown to activate different UPR signaling pathways and preferentially utilize these pathways to promote their replication. Infections with the mosquito-borne flaviviruses (MBFV) dengue virus (DENV), Japanese encephalitis virus (JEV), West Nile virus (WNV), and Zika virus (ZIKV) as well as infection with the tick-borne flavivirus (TBFV) Langat virus (LGTV) have been demonstrated to lead to an increased expression of BiP [14,15,17,22,23]. ZIKV, DENV and WNV activate all three UPR pathways [14,17,18]. IRE1 promotes ZIKV infection and both, IRE1 activity as well as spliced XBP1, were required for optimal virus RNA replication [24]. In DENV infection, PERK exhibits an antiviral role, IRE1 facilitates virus replication and ATF6 has no effect on virus replication [16]. In WNV-infected cells, IRE1 and PERK signaling have no effect on virus replication [19], whereas ATF6 supports virion production [14]. The related but low pathogenic Usutu virus (USUV) was found to induce the IRE1 pathway by XBP1 splicing [18]. Furthermore, JEV is known to induce IRE1 signaling with beneficial effects on JEV replication [20]. LGTV and the closely related but more pathogenic Powassan virus (POWV) induce PERK signaling. PERK knockdown resulted in increased LGTV replication but had no effect on POWV infectious titers suggesting a strategy to antagonize PERK-mediated antiviral effect [15]. TBEV was shown to induce the ATF6 and IRE1 pathways with IRE1 favoring viral replication [25].

Analysis of UPR signaling between different viral strains and between closely related viruses (e.g., different DENV serotypes, WNV NY99 versus WNV KUN, WNV versus USUV, and POWV versus LGTV) clearly illustrates how viruses of various pathogenicity differentially activate and utilize UPR pathways [14,15,20,21,23]. One reason for this diverse outcome may lie in the manipulation of the host cell machinery to a different extent by viruses of different pathogenicity but also in the design of the studies itself. Most of the studies have been conducted in nonrelevant and varying cell lines that do not coincide with the naturally observed tropisms of the flaviviruses [14,15,16,17,18,21,24,25], complicating a comprehensive comparison of UPR activation between viruses. The aim of our study was to analyze and compare the activation as well as the role of the UPR pathways in TBFV infections of different pathogenicity in relevant viral target cells. TBEV strain Neudoerfl was isolated from a tick in Austria and has served as a prototype of a neurotropic Central European TBEV isolate that has been studied extensively in animal models. In contrast, TBEV strain MucAr-HB-171/11 was isolated from a tick from a natural focus, in which human infections with atypical symptoms, such as mostly mild gastrointestinal disease, had occurred. Despite its close phylogenetic relationship to Czech neurotropic TBEV strains, it was confirmed to differ in neuroinvasiveness and virulence in a mouse model [4]. LGTVwas isolated from ticks in Malaysia and found to be naturally attenuated. However, attempts to use LGTV as a vaccine strain failed when 1 out of 10,000 study participants developed neurological disease [26].

## 2. Materials and Methods

### 2.1. Cell Culture

Human astrocytoma cells (SNB19, ATCC HTB-14, American Type Culture Collection, Manassas, VA, USA), human intestinal epithelial cells (Caco2, ATCC HTB-37, American Type Culture Collection, Manassas, VA, USA), human lung epithelial cells (A549, ATCC CCL-185, American Type Culture Collection, Manassas, VA, USA), and African green monkey epithelial kidney cells (Vero E6, ATCC CRL-1586, American Type Culture Collection, Manassas, VA, USA) were maintained in high-glucose Dulbecco’s modified Eagle’s medium (DMEM) (Sigma-Aldrich, Taufkirchen, Germany) supplemented with 10% fetal calf serum (FCS), 1% penicillin (10,000 U/mL)/streptomycin (10 mg/mL) solution, as well as 2 mM L-glutamine solution (all purchased from Sigma-Aldrich, Taufkirchen, Germany) at 37°C in 5% CO_2_. Human neuroblastoma cells (SH-SY5Y, ATCC CRL-2266, American Type Culture Collection, Manassas, VA, USA) were proliferated in high-glucose DMEM containing 15% FCS, 1% pen/strep solution, and 4 mM L-glutamine at 37 °C in 5% CO_2_. For differentiation, SH-SY5Y cells were incubated with high-glucose DMEM supplemented with 1% pen/strep solution, 1 mM L-glutamine, 1 mM sodium pyruvate and 10 µM retinoic acid at 37 °C in 5% CO_2_ for 6 days.

### 2.2. Virus Strains

All virus strains were obtained from the collection of the Department of Microbiology of the German Armed Forces in Munich, Germany. TBEV strains Neudoerfl (GenBank accession number U27495) and MucAr-HB-171/11 (GenBank accession number KX268728, from here on referred to as TBEV HB171) were propagated in A549 cells. LGTV (GenBank accession number AF253419) was propagated in Vero E6 cells. SNB19 cells were seeded 1 day prior to infection at a density of 1–2 × 10^5^ cells/mL depending on the culture format. SH-SY5Y were seeded 7 days prior to infection at a density of 0.2–1 × 10^6^ cells/mL in proliferation medium and differentiated for 6 days starting from 1 day post seeding. Caco2 cells were seeded 4 days prior to infection at a density of 0.5–2 × 10^5^ cells/mL depending on the culture format. For infections, cells were counted and incubated with virus at a multiplicity of infection (MOI) of 0.1 for one hour before they were washed with phosphate buffered saline (PBS) to remove unbound virus particles and further incubated with DMEM containing 2% FCS, which marks the time point 0 h.

### 2.3. Antibodies for Immunoblotting

The antibodies used for immunoblotting were rabbit (rb) anti-BiP (Cell Signaling, #3177), rb anti-XBP1s (Cat. #40435, Cell Signaling, Frankfurt, Germany), rb anti-ATF6 (Cat. #65880, Cell Signaling, Frankfurt, Germany), rb anti-eIF2α (Cat. #5324, Cell Signaling, Frankfurt, Germany), rb anti-p-eIF2α (Cat. #9721, Cell Signaling, Frankfurt, Germany), rb anti-GAPDH (Cat. #5174, Cell Signaling, Frankfurt, Germany) and HRP-conjugated goat anti-rb-IgG secondary antibody (Cat. #SBA-4030-05, Biozol, Eching, Germany).

### 2.4. Protein Analysis

Cells were washed with PBS and lysed for 1 h at room temperature (RT) in lysis buffer (25 mM Tris, 50 mM NaCl, 0.5% Triton X-100, 0.5% sodium deoxycholate, pH 7.4, protease inhibitor cocktail 1:50, all purchased from Carl Roth, Karlsruhe, Germany). After addition of 2 × loading buffer (50 mM Tris HCl (pH 6.8), 10% glycerol, 2% SDS, 0.02% bromophenol blue, 100 mM DTT), the samples were denatured for 5 min at 95°C. Proteins were separated by sodium dodecyl sulfate polyacrylamide gel electrophoresis (SDS-PAGE) and transferred onto a nitrocellulose membrane (Carl Roth, Karlsruhe, Germany) using a wet-blotting chamber (Bio-Rad, Munich, Germany). Membranes were blocked in 5% low fat milk powder in PBS + 0.05% Tween 20 (PBS-T) for one hour at RT and incubated with primary antibodies (BiP, ATF6, XBP1s, eIF2α, p-eIF2α, GAPDH) at 1:5000 dilution and 4 °C overnight, followed by the appropriate HRP-conjugated secondary antibody (1:5000 dilution) for one hour at RT. The chemiluminescent signal was developed using SuperSignal West Femto Maximum Sensitivity Substrate (Thermo Fisher, Dreieich, Germany) and detected in the Chemidoc system (Bio-Rad, Munich, Germany). For stripping, membranes were washed with PBS-T for 15 min and incubated in 200 mM glycine, 3.5 mM SDS, 1% Tween 20, pH 2.2 for one hour.

### 2.5. Intracellular mRNA Analysis

Infected cells were lysed by Trizol (Thermo Fisher, Dreieich, Germany) and cellular RNA was extracted using the Direct-zol RNA kit (Zymo Research, Freiburg, Germany), followed by reverse transcription to cDNA (ProtoScript II, New England Biolabs, Frankfurt, Germany). PCR detection of unspliced and spliced XBP1 was performed as described by Samali et al. [27]: forward primer 5′-TTACGAGAGAAAACTCATGGCC-3′ and reverse primer 5′-GGGTCCAAGTTGTCCAGAATGC-3′. The housekeeping gene glyceraldehyde-3-phosphate dehydrogenase (GAPDH) was used as a loading control as described by Kurisaki et al. [28]: forward primer 5′-CCCATGTTCGTCATGGGTGT-3′ and reverse primer 5′-TGGTCATGAGTCCTTCCACGATA-3′. The amplified gene products were separated by electrophoresis on a 2% agarose gel and visualized by the Chemidoc system (Bio-Rad, Munich, Germany).

### 2.6. IRE1 Inhibition

To inhibit IRE1, cells were treated with the inhibitors KIRA6, STF083010 and GSK2850163 (all purchased from Sigma-Aldrich, Taufkirchen, Germany). SNB19 and SH-SY5Y cells were pretreated for 4 h with 100 nM KIRA6, 50 µM STF083010 or 500 nM GSK2850163. To inhibit IRE1 in Caco2 cells 300 nM KIRA6, 100 µM STF083010 and 1 µM GSK2850163 were used. Subsequently, cells were incubated with virus at MOI 0.1 for another hour, before they were washed with PBS to remove unbound virus particles and further incubated with DMEM containing 2% FCS and the respective inhibitor. Every 24 h, starting directly after removing the virus inoculum and ending at 72 h post infection (hpi), cells were either lysed in lysis buffer for subsequent protein analysis or resuspended in Trizol (Thermo Fisher, Dreieich, Germany) for mRNA extraction (see methods above). Further, cell culture supernatant containing virus particles was collected at all time points for viral RNA extraction (Viral RNA Mini kit, Qiagen, Hilden, Germany) and quantification by probe-based quantitative reverse transcription (RT)-PCR as described for TBEV by Schwaiger and Cassinotti [29] and for LGTV by Kurhade et al. [30].

### 2.7. Cell Viability

IRE1 inhibitors were titrated on each cell line to determine the non-toxic range in which cell viability is not affected by the inhibitor. Cell viability in the presence of inhibitor was measured using the CellTiter Glo assay kit (Promega, Walldorf, Germany) and a multiwell plate reader (Tecan, Crailsheim, Germany).

### 2.8. Statistical Analysis

The results were statistically analyzed by GraphPad Prism 9.0.0 (GraphPad Software, San Diego, CA, USA). Two-way ANOVA with Tukey’s multiple comparison test was performed to compare the virus replication within different cell lines and between untreated and treated cells.

## 3. Results

### 3.1. Viral Replication Efficiency in CNS or Intestinal Cells Varies by TBFV Strain

**Figure 1:**
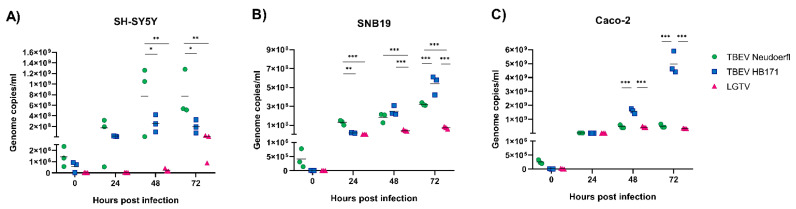
Comparison of the replication efficiencies of the different tick-borne flaviviruses in neuronal (**A**), astrocyte (**B**) and intestinal (**C**) cell lines. Cells were infected at multiplicity of infection of 0.1 and supernatants were collected at 0, 24, 48 and 72 h post infection. Viral RNA was extracted from culture supernatants and quantified by qRT-PCR. Genome copy numbers of Neudoerfl are shown in green, HB171 in blue and LGTV in pink. All infections were performed in triplicates and repeated three times. Statistical analyses were performed by 2-way ANOVA with Tukey’s multiple comparison test, * *p* < 0.033, ** *p* < 0.002, *** *p* < 0.001.

To compare the replication efficiencies of TBEV Neudoerfl, TBEV HB171 and LGTV in the three different cell lines SH-SY5Y, SNB19 and Caco-2, cells were infected with the three viruses at MOI 0.1 and monitored for genomic viral RNA in culture supernatants for up to 72 h post infection. The neurotropic TBEV strain Neudoerfl replicated to significantly higher titers in the neuronal SH-SY5Y cells than the other TBFV, though this became apparent only at later time points of infection (Figure 1A). In contrast, TBEV strain HB171, which is associated with gastrointestinal symptoms, replicated to significantly higher titers in the intestinal cell line Caco-2 at later stages of infection when compared to the other TBFV (Figure 1C). The data reflect the observed natural tropism of the two TBEV strains. In the astrocytoma cell line SNB19 and the neuronal SH-SY5Y cells, LGTV grew to significantly lower titers than the more pathogenic TBEV strains (Figure 1A,B). The differences between Neudoerfl and HB171 in astrocytoma cells were less clear (Figure 1B). While Neudoerfl yielded significantly higher titers than HB171 at the early stages of infection, HB171 infections led to the highest titers at later time points (Figure 1B). It was also apparent that TBEV strain Neudoerfl replicated to one-log higher titers throughout the time course of infection in CNS-resident cells than in intestinal cells (Figure 1A–C). In contrast, LGTV replication was delayed in neuronal SH-SY5Y cells (Figure 1A).

### 3.2. The UPR Is Activated by TBFV Infection of CNS and Intestinal Cells

**Figure 2:**
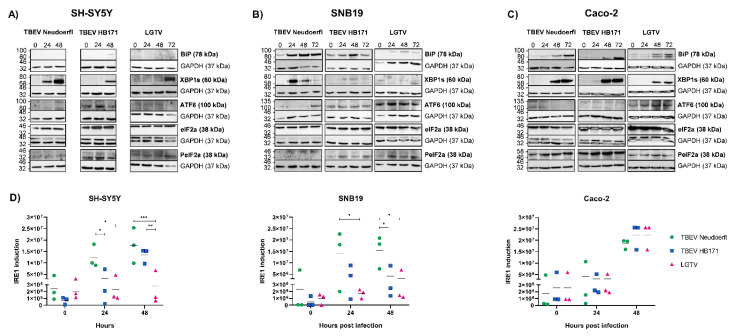
Induction of three different pathways of the unfolde protein response by the different tick-borne flaviviruses in neuronal (**A**), astrocyte (**B**) and intestinal (**C**) cells. Cells were infected at multiplicity of infection of 0.1 and cell lysates were collected at 0, 24, 48 and 72 h post infection. The lysates were analyzed by SDS-PAGE and Western blot probed for proteins of the IRE1 pathway (GRP78/BiP, XBP1), the ATF6 pathway (ATF6) or the PERK pathway (eIF2α, p-eIF2α). The experiments were repeated three times and a representative blot is shown. GAPDH was included as a housekeeping control. Asterisks (*) indicate unspecific bands in GAPDH controls for eIF2α due to incomplete stripping of the membrane and presence of residual eIF2α signal. (**D**) Quantification of XBP1 Western blot signal intensities of three independent experiments and comparison of XBP1 protein levels in the three tested cell lines infected with each virus. Statistical analyses were performed by 2-way ANOVA with Tukey’s multiple comparison test, * *p* < 0.033, ** *p* < 0.002, *** *p* < 0.001. kDa, kilodalton.

Next, we analyzed the lysates of infected cells for the expression of different cellular factors that are associated with the three different UPR pathways, namely GRP78/BiP as a general ER stress marker, XBP1 for the IRE1 pathway, ATF6 for the ATF6 pathway and eIF2α or p-eIF2α for the PERK pathway. While UPR induction with drugs, such as tunicamycin or thapsigargin, led to similar levels of XBP1 expression in the tested cell lines, the kinetics were much faster than for TBEV infection, leading to high levels of XBP1 expression within hours rather than days (data not shown). For this reason, drug treatments have not been included as positive control in the infection experiments. Interestingly, very little BiP could be detected in neuronal SH-SY5Y cells upon infection with any of the viruses (Figure 2A). The neuronal SH-SY5Y cells did not survive infection with either TBEV strain for up to 72 h in most experiments, whereas infection with LGTV was tolerated for longer time frames due to the delayed replication kinetics. Thus, UPR-associated protein expression in SH-SY5Y cells upon TBEV infection was only measured up to 48 h post infection (Figure 2A). The IRE1 activation marker XBP1 was strongly induced in neuronal SH-SY5Y and intestinal Caco-2 cells upon infection with all three TBFV and in astrocytoma SNB19 cells upon infection with TBEV Neudoerfl (Figure 2A–C). In contrast, the ATF6 and PERK pathways appear to play only a minor role in TBFV infection of the tested cell lines. Both pathways were not activated in neuronal SH-SY5Y cells upon infection with TBEV Neudoerfl or LGTV, but were induced at 24 h in TBEV HB171 infected cells with ATF6 and p-eIF2α levels detectable even earlier than XBP1 in these cells (Figure 2A). A late induction of the ATF6 pathway could be observed for TBEV Neudoerfl infected astrocytoma SNB19 cells at 72 h post infection. However, proteolytically activated ATF6 was not detected in any of the samples (data not shown). Low-level phosphorylation of eIF2α was detectable in SNB19 cells infected with TBEV HB171 and LGTV starting at 24 h post infection (Figure 2B). Finally, in intestinal Caco-2 cells a low-level induction of ATF6 was detectable in LGTV infected cells (Figure 2C). Signaling proteins that were detectable at 0 h post infection were considered unrelated to the virus infection and are not discussed here. Our data indicate that IRE1 activation, leading to the expression of the active transcription factor XBP1, may be the most distinct UPR pathway in TBFV infection of the relevant target cells.

### 3.3. TBFV-Induced IRE1 Activation Differs between Cell Types and Viral Strains

When we compared IRE1 activation between cell types and viral strains, we found that the kinetics of the induction of this pathway in SH-SY5Y cells varied substantially between different TBFV strains. While XBP1 was readily detectable after 24 h in TBEV Neudoerfl infected cells and peaked at 48 h post infection, XBP1 became apparent only at 48 h post infection in TBEV HB171-infected cells (Figure 2A). LGTV induced XBP1 expression even later at 72 h post infection, which could be explained by the delay in replication (Figure 1A). As SH-SY5Y cells showed extensive cytopathic effects at 72 h post infection with the two TBEV strains in some experiments, data from the late stages of infection have been excluded from the analysis (Figure 2A). In astrocytoma SNB19 cells, differences between viral strains became even more apparent. BiP was strongly induced by infection with TBEV Neudoerfl, but to a lesser extent with the other two TBFV (Figure 2B). Similarly, the IRE1 activation marker XBP1 was expressed at high levels in SNB19 cells at 24 and 48 h post infection with TBEV Neudoerfl, but was only mildly induced in cells infected with the other two TBFV (Figure 2B). The strong reactions of neuronal SH-SY5Y and astrocytoma SNB19 cells to infection with the neurotropic TBEV Neudoerfl suggests a possible role of this mechanism in CNS-resident cells in viral pathogenesis. The induction of the IRE1 activation marker XBP1 in intestinal Caco-2 cells appeared earlier after infection with TBEV HB171 with a protein band becoming visible already at 24 h post infection (Figure 2C), which correlates with the naturally observed tropism of this virus strain. Quantification of XBP1 Western blot signal intensities from three independent experiments revealed differences between the three virus strains tested on the three cell lines (Figure 2D). While there was some variation between individual experiments, XBP1 protein levels were consistently lower in SNB19 cells infected with TBEV HB171 or LGTV as compared to TBEV Neudoerfl (Figure 2B,D). Similar patterns were observed in SH-SY5Y cells, whereas no clear differences could be observed in Caco-2 cells (Figure 2D).

### 3.4. Unconventional Splicing of XBP1 Confirms Activation of IRE1 Signaling by TBFV Infection

**Figure 3:**
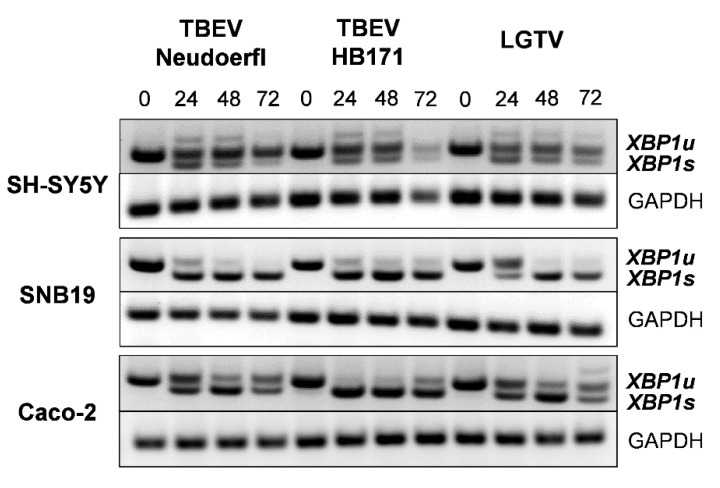
Analysis of XBP1 mRNA splicing by IRE1 after infection of neuronal (SH-SY5Y), astrocyte (SNB19) and intestinal (Caco-2) cells with the different tick-borne flaviviruses. Cells were infected at multiplicity of infection of 0.1 and cell lysates were collected at 0, 24, 48 and 72 h post infection. Cellular RNA was extracted from lysates, quantified by RT-PCR and analyzed on an agarose gel. Spliced XBP1 (XBP1s) can be distinguished from unspliced XBP1 (XBP1u) by a shift towards a lower molecular weight. GAPDH was used as a housekeeping control. The experiment was repeated three times and a representative gel is shown.

As IRE1 activation was found to be important in TBFV infection, we sought to confirm our observation on the level of XBP1 mRNA. The infection of all three cell lines with the three TBFV was performed as before, but cell lysates were harvested at different time points for the extraction of mRNA, RT-PCR and subsequent detection of spliced and unspliced forms of XBP1 cDNA. Our PCR data were consistent with an induction occurring at the protein level. While XBP1 mRNA unconventional splicing in neuronal SH-SY5Y cells was similar after infection with both TBEV strains, it was faster and more pronounced in Caco-2 cells upon infection with TBEV HB171 (Figure 3). Interestingly, despite the low level of XBP1 protein induction in astrocytoma SNB19 cells after infection with TBEV HB171 and LGTV, XBP1 mRNA splicing seemed to occur at a similar level compared to TBEV Neudoerfl infected cells, though with slower kinetics for LGTV (Figure 3). The reasons behind this discrepancy are currently unclear. It is possible that mRNA splicing occurs at a different time scale compared to protein expression. Moreover, any quantitative differences in levels of transcripts will be lost during PCR amplification and would not be detected here.

### 3.5. Inhibition of the IRE1 Pathway Leads to a Reduction of Virus Replication in Astrocytoma Cells

**Figure 4:**
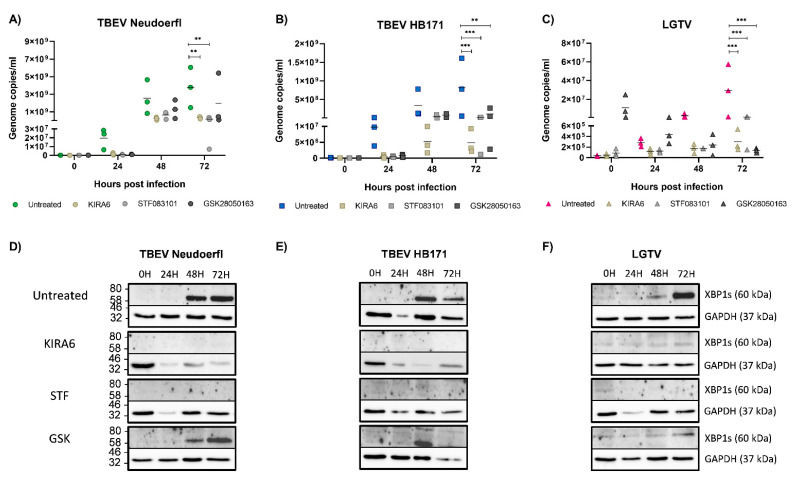
Infection experiments with the neuronal cell line SH-SY5Y were repeated as described in Figure 1, but cells were pretreated with IRE1 inhibitors KIRA6 (KIRA), STF083010 (STF) and GSK2850163 (GSK) for four hours prior to infection as well as after infection or left untreated. Viral RNA was extracted from culture supernatants and cell lysates were collected at 0, 24, 48 and 72 h post infection. Infections of untreated cells with Neudoerfl are shown in green (**A**), HB171 in blue (**B**) and LGTV in pink (**C**). Infections of cells pretreated with KIRA are shown in light brown, STF in light grey and GSK in dark grey, respectively. All infections were performed in triplicates and repeated three times. Statistical analyses were performed by 2-way ANOVA with Tukey’s multiple comparison test, ** *p* < 0.002, *** *p* < 0.001. Cell lysates were analyzed by Western blot for XBP1 expression after infection with Neudoerfl (**D**), HB171 (**E**) or LGTV (**F**). GAPDH expression was analyzed as a housekeeping control. A representative blot is shown. kDa, kilodalton.

**Figure 5:**
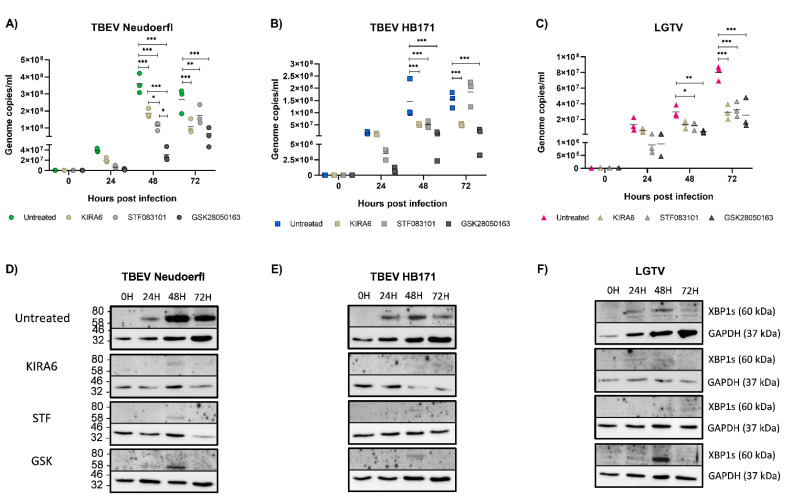
Infection experiments in the astrocyte cell line SNB19 were performed as described in Figure 4. Infections of untreated cells with Neudoerfl are shown in green (**A**), HB171 in blue (**B**) and LGTV in pink (**C**). Infections of cells pretreated with KIRA are shown in light brown, STF in light grey and GSK in dark grey, respectively. All infections were performed in triplicates and repeated three times. Statistical analyses were performed by 2-way ANOVA with Tukey’s multiple comparison test, * *p* < 0.033, ** *p* < 0.002, *** *p* < 0.001. Cell lysates were analyzed by Western blot for XBP1 expression after infection with Neudoerfl (**D**), HB171 (**E**) or LGTV (**F**). GAPDH expression was analyzed as a housekeeping control. A representative blot is shown. kDa, kilodalton.

**Figure 6:**
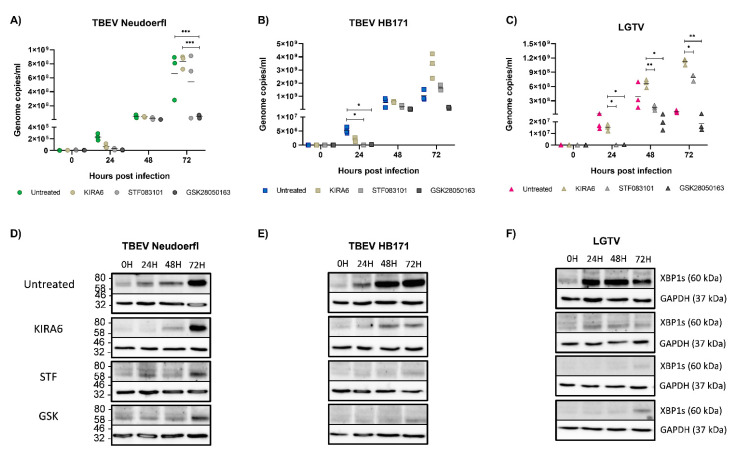
Infection experiments in the intestinal cell line Caco-2 were performed as described in Figure 4. Infections of untreated cells with Neudoerfl are shown in green (**A**), HB171 in blue (**B**) and LGTV in pink (**C**). Infections of cells pretreated with KIRA are shown in light brown, STF in light grey and GSK in dark grey, respectively. All infections were performed in triplicates and repeated three times. Statistical analyses were performed by 2-way ANOVA with Tukey’s multiple comparison test, * *p* < 0.033, ** *p* < 0.002, *** *p* < 0.001. Cell lysates were analyzed by Western blot for XBP1 expression after infection with Neudoerfl (**D**), HB171 (**E**) or LGTV (**F**). GAPDH expression was analyzed as a housekeeping control. A representative blot is shown. kDa, kilodalton.

To investigate the role IRE1 induction may play for virus replication in the three different cell lines, we used three IRE1 inhibitors with different mechanisms blocking either the kinase activity of IRE1 (KIRA6) or the endonuclease activity (STF083010) or both (GSK2850163). Non-toxic concentrations of the inhibitors were independently determined for each cell line by incubation with serial dilutions of the inhibitors for up to 72 h and subsequent measurement of cell viability (data not shown). Cells were pretreated for four hours prior to infection to ensure the inhibitors took effect by the time of infection. In neuronal SH-SY5Y cells, the inhibitors KIRA6 and STF083010 were able to completely block the induction of XBP1, while residual protein was detected after treatment with GSK2850163 (Figure 4D–F). Inhibition of IRE1 in neuronal SH-SY5Y cells led to a reduction of viral replication for all three viruses, though differences were not significant (Figure 4A–C). In contrast, in astrocytoma SNB19 cells, viral replication was reduced significantly with the most pronounced effects observed for GSK2850163, despite the detection of residual XBP1 at later time points (Figure 5A–F). The dual inhibitor also had an effect on viral replication in Caco-2 cells, though the differences in viral titers were lost at later time points (Figure 6A–C). Despite the use of higher inhibitor concentrations in the more robust Caco-2 cell line, more residual XBP1 protein was detected after inhibitor treatment than with the other cell lines, especially at later time points, indicating a limited half-life of the compounds (Figure 6D–F). Inhibition of the kinase activity of IRE1 effectively led to an increase in viral replication of LGTV (Figure 6C). However, our data indicates a supporting role of the IRE1 pathway for TBFV infection in CNS cells as evidenced by a reduction in viral replication upon complete or partial inhibition of this UPR pathway in CNS-resident cells. Limitations of our study are given by the narrow therapeutic indices of the IRE1 inhibitors and the delicate nature of the neuronal cell cultures. A reduction in signal for housekeeping proteins in some samples suggests that the inhibitors may also affect other metabolic pathways.

## 4. Discussion

In the present study, we describe a differential pattern of UPR activation by two strains of TBEV in CNS cells. This suggests that UPR activation may be involved in the immune response against viral infection and neuroinflammation observed in TBEV infection of the CNS. Different observations have been made in previous studies on UPR activation by TBEV and related flaviviruses [15,17,18,19,20,23,24,25]. While all studies supported a role of the UPR in flavivirus infection, the signaling pathways implicated differed. Thus, the PERK pathway has been associated with cell survival and extended viral replication in DENV-2-infected canine kidney MDCK or mosquito C6/36 cells [31,32]. By contrast, this pathway mediates apoptosis in JEV-infected mouse neuronal Neuro-2a cells [33] and restriction of LGTV replication in human embryonal kidney 293T cells [15]. This suggests a virus and cell-type dependent activation of the three arms of the UPR. Additionally, a time-dependent activation of the different pathways throughout consecutive stages of DENV-2 infection has been demonstrated [16], adding another layer of differential regulation of cellular stress responses by flaviviruses. In the present study, we found little evidence of PERK induction upon TBFV infection of the relevant target cells with only a minor increase in phosphorylated eIF2α in astrocytoma cells.

The two other arms of the UPR, the IRE1α and ATF6 pathways, have mostly been implicated in the restoration of cellular homeostasis under stress conditions [34] and, in the context of infection, may play a supportive role in viral protein production. While ATF6 has mainly been implicated in ER quality control, IRE1 has been described to have an extended role in protein synthesis and transport [34]. A knock-out of ATF6 had no effect on DENV replication [16]. In contrast, WNV production decreased in the absence of ATF6 [14]. ATF6 has been shown to be upregulated in nervous tissue in a mouse model for ZIKV infection and likely contributes to neuropathogenesis [23]. Moreover, Yu et al. observed the proteolysis and translocation of ATF6 during TBEV infection of human embryonic kidney cells [25]. In our study, the ATF6 signaling pathways seemed to play a minor role in TBFV infection. An increase of full-length ATF6 protein was detected at late time points in neurotropic TBEV Neudoerfl infection of astrocytoma cells and throughout the course of LGTV infection of intestinal cells. However, no proteolytic cleavage or translocation of activated ATF6 could be observed. Furthermore, based on our data, ATF6 does not play a role in UPR induction in neuronal cells.

Our study of TBFV-mediated UPR activation in relevant viral target cells identified the IRE1-XBP1 axis as the dominant pathway. The strongest indicator of IRE1 pathway activation is unconventional splicing and expression of the active XBP1s transcription factor [35]. XBP1s in turn regulates processes associated with modulation of ER function, organelle expansion, and energy metabolism [36]. Additional XBP1s target genes include ERAD components that reduce the protein burden by targeted proteolysis and cellular factors required for protein trafficking to the Golgi [37]. Besides XBP1, IRE1 cleaves also other mRNAs to limit translocation to the ER through regulated IRE1-dependent decay (RIDD) [38]. By association with TRAF2, IRE1 activation further channels into innate immune pathways via NFκB and JNK signaling leading to inflammation and apoptosis [39]. Situated at the crossroads between mRNA and protein degradation, maintenance of cellular homeostasis and innate immune signaling, the IRE1 arm of the UPR dictates cell fate. Therefore, it is not surprising that viruses interact with and manipulate this pathway. For many flaviviruses, IRE1 pathway activation has been described to play a supportive role by promoting biogenesis and secretion of virions [14,17,18,22,24,25]. In fact, the characteristics of a flavivirus infected cell with largely expanded ER, activated IRE1 and upregulated XBP1s, resemble the environment of secretory cells, such as plasma cells, whose increased production of large amounts of IgG induces ER stress and activates the UPR [35,40]. This may be explained by the similar demands put on the biosynthetic and secretory pathways of the infected cell by viral replication, assembly, budding and release of virions. In the CNS, dysregulation of IRE1 signaling is implicated in a number of neurodegenerative disorders [41]. It has been suggested that neuroinflammation following infection with neurotropic viruses may play a role in these mechanisms [42,43,44,45,46]. Our observation that IRE1 activation in astrocytoma cells was stronger for the neurotropic pathogenic TBEV strain Neudoerfl, suggests that immune-mediated pathomechanisms may contribute to the severe form of the disease that is not observed for the other two viruses. Furthermore, the different kinetics of virus replication and IRE1 activation in neuronal cells indicate a heavier protein biosynthesis burden in cells infected with TBEV strain Neudoerfl. This goes in line with IRE1 inhibition having the strongest effect on viral replication in astrocytoma cells. The lack of a full inhibition of viral replication by any of the tested inhibitors suggests a supporting but non-essential role of IRE1 signaling for viral replication.

Differences in UPR signaling between diverse viral strains and closely related viruses demonstrate how viruses of varying pathogenicity activate and exploit the UPR pathways in distinct ways [3,8,15,33]. In our study, we focused on TBFV with differing pathogenic potential and performed our analysis of the UPR pathways in relevant human target cells of TBEV infection. The replication efficiency and ability to induce the UPR in these cells seem to correlate with the naturally observed tropism of the virus strains. This hints at the UPR as an important mechanism for TBFV replication that could be used to judge the pathogenic potential of different virus strains and represents a possible target for therapeutic intervention. In fact, ER stress responses are targeted in a number of completed and ongoing clinical trials for metabolic diseases, such as diabetes, cancer, vascular disease and immune function in yellow fever virus infection (e.g., available online: https://clinicaltrials.gov (last accessed 21 October 2021): NCT02368704, NCT00773747, NCT04001647, NCT04267809). Based on our data, the impact of TBEV-mediated IRE1 induction in astrocytes on immune signaling and the protection of neurons presents an interesting objective for further studies. Which genetic changes between TBEV strains Neudoerfl and HB171 may be involved in the observed differences in UPR induction (and variation in neuroinvasion and virulence described by others) is at present unclear and will be the subject of future studies.
